# Left renal failure caused by chronic obstructive uropathy due to a uretero-inguinal hernia combined with contralateral renal pelvic carcinoma: A case report

**DOI:** 10.1097/MD.0000000000009133

**Published:** 2017-12-22

**Authors:** Weiling Xu, Fangchao Gong, Dong Dong, Baorong Chi, Jingyu Wang

**Affiliations:** a Department of Radiology, The First Hospital of Jilin University; b Department of Surgery, The First Hospital of Jilin University; c Department of Intern, The First Hospital of Jilin University, Changchun, China.

**Keywords:** CT, renal failure, renal pelvic carcinoma, uretero-inguinal hernia

## Abstract

**Rationale:**

Although rare, cases of renal failure secondary to a uretero-inguinal hernia have been reported.

**Patient concerns:**

Here, we report a case of left renal failure caused by chronic obstructive uropathy due to a uretero-inguinal hernia combined with contralateral renal pelvic carcinoma.

**Diagnoses:**

The present case highly indicated that a comprehensive examination is very important when diagnosing an inguinal hernia. In particular, it is necessary to check whether the ureter is involved or not.

**Interventions:**

A computed tomography scan should be performed to a uretero-inguinal hernia patient.

**Outcomes:**

Unfortunately, this patient was diagnosed too late to attempt surgical management to restore the left renal functions.

**Lessons:**

In our opinion, a computed tomography scan is highly recommended for an accurate diagnosis.

## 1 Introduction

A ureteral hernia, a peculiar form of a sliding inguinal hernia, occurs infrequently.^[1]^ In addition, it may be difficult to detect because there are no symptoms at the early stage. Unfortunately, the ignored uretero-inguinal hernia will gradually progress to obstructive uropathy, and then lead to chronic hydronephrosis and kidney injury.^[2]^ In clinical practice, the diagnosis of a uretero-inguinal hernia is usually made by imaging examinations, such as a computed tomography (CT) scan of the abdomen and/or pelvis.^[3]^ Here, we report an extremely rare case of a uretero-inguinal hernia, which led to hydronephrosis and renal failure, and contralateral renal pelvic carcinoma.

## 2 Case

The participant signed an informed consent form prior to the initiation of this study, and all study procedures were approved by the Ethical Committee of the First Hospital of Jilin University.

A 70-year-old man was admitted to the hospital due to the presence of a mass for more than 2 years without an imaging examination, complaint of gross hematuria for 1 month, and aggravation for 3 days. He had no previous surgeries, and his body mass index was 20.2. Upon physical examination, a mass in the left scrotum was discovered, which was painless, originated above the inguinal ligament, and could be returned to the abdominal cavity. The results of routine serum analysis were as follows: red blood cell count, 29.0/μL and 4.8/high power field; white blood cell count, 91.0/μL and 15.2/high power field. There were no abnormalities in terms of tumor markers, serum creatinine levels, or blood urea nitrogen levels.

**Figure 1:**
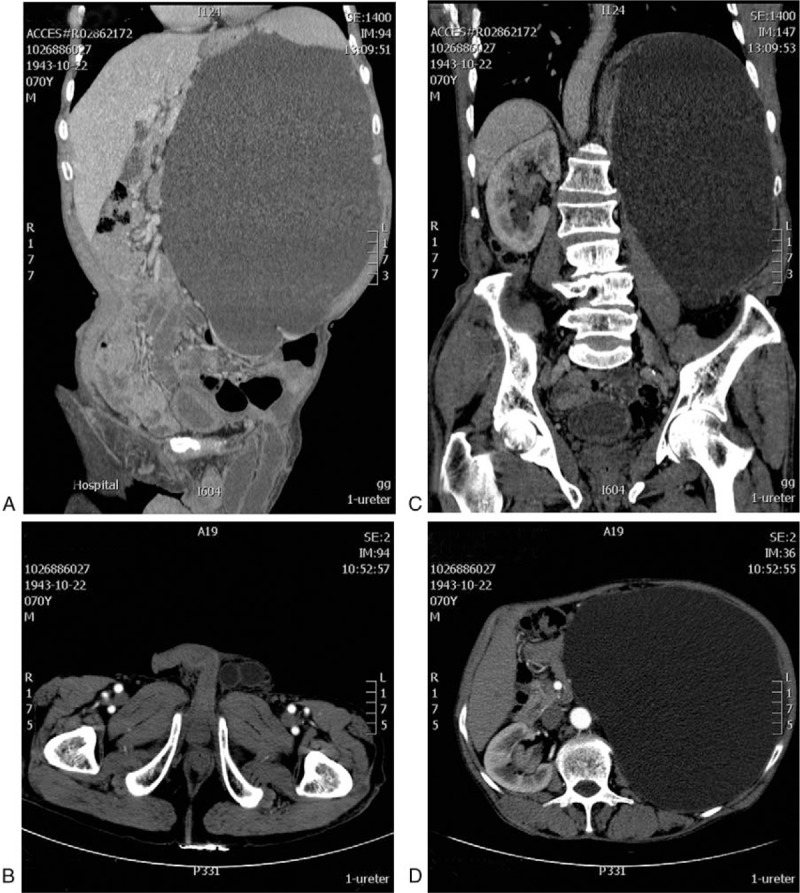
CT images of a 70-year-old patient with a left uretero-inguinal hernia, left hydronephrosis, and right renal pelvic carcinoma. (A) The coronary CT shows a ureteral loop in the inguinal canal and severe hydronephrosis of the left kidney. (B) The dilated ureter in the inguinal canal is shown on the axial CT. The coronary (C) and axial CT (D) show the dilated pyelocaliceal system and a mass in the right renal pelvis. CT = computed tomography.

Ultrasonography showed a severe left uretero-hydronephrosis, with thickness of the right renal pelvis wall. In addition, a CT scan including the abdomen and pelvis was performed, which revealed a left hyperdilated extrarenal pelvis with a dilated ureter and a left inguinal hernia including the ureter (Fig. 1A and B). No enhancement was found in the left kidney after contrast agent administration, while strong enhancement was observed in the right thickened renal pelvic wall (Fig. 1C and D), which indicated the possibility of carcinoma. Considering the contraindication of surgery, a diagnosis of right renal pelvic carcinoma was established based on the CT scan. After undergoing percutaneous nephrostomy of the left kidney, he was discharged, waiting for a possible kidney transplant. During the follow-up process, the patient withdrew from treatment due to personal reasons.

## 3 Discussion

Although rare, cases of renal failure secondary to uretero-inguinal hernia have been reported previously in the literature.^[4]^ For example, an achondroplastic male experienced acute kidney injury due to bilateral hydronephrosis and ureteric obstruction secondary to bilateral uretero-inguinal herniation.^[5]^ Recently, a rare case with a uretero-inguinal hernia and a pelvic mass on the right side has also been reported, and this patient was successfully treated with a right nephroureterectomy and inguinoscrotal hernia repair.^[6]^ In the present case, a left uretero-inguinal hernia led to severe hydronephrosis and renal failure, and a contralateral renal pelvic carcinoma was also detected. We hypothesized that there is no inherent correlation between the incidences of these 2 diseases. It is certain that relief of the ureteral obstruction could restore kidney function in acute kidney failure, while our case presented with chronic kidney injury and obvious atrophy of the renal parenchyma as observed in the CT scan. Therefore, the left renal function was poor and could not be restored by relieving the ureteral obstruction. Although the creatinine and blood urea nitrogen levels were within the normal range, the patient would require renal replacement therapy if the right kidney was resected.

Even though involvement of the ureter in inguinal hernias is rare, it causes great harm to the patient's health, especially in delayed diagnosis. Imaging examinations on hernias may provide the most important diagnostic evidence. Among the present imaging examination techniques, CT can not only present the morphological features of the covered range, but it also can check the function of the involved kidney after the application of contrast agent.

Unfortunately, the uretero-inguinal hernia was not found until the left kidney function had been lost. Besides, an additional mass in the right renal pelvis was also found. Under these circumstances, surgical removal of the right pelvic mass was contraindicated and kidney transplantation was highly recommended.

In conclusion, the present case highly indicated that comprehensive examination is very important when diagnosing an inguinal hernia. In particular, it is necessary to check whether the ureter is involved or not. In our opinion, a CT scan is highly recommended for an accurate diagnosis.
